# Chloral in Obstetrics

**Published:** 1871-05

**Authors:** 


Chloral in Obsetrics.—Dr. S. F. Starley, of Fairfield, Texas, writes as follows concerning the value of *chloral* in obstetric practice:

"For nearly twenty years I have used chloroform in parturition; but since the introduction of chloral I have substituted it for the former in a number of labors, and find it a valuable anæsthetic, and in many respects greatly preferable to chloroform. It is easy of administration, does not require the constant attention of the accoucher, and produces no sudden or overwhelming effect. I usually commence with twenty grains; in one or two hours, fifteen to twenty grains again, and then often enough to keep up the anæsthetic effect. Quite recently I was called to a lady in her third labor, who, from living in a malarial region, and from repeated chills, had well-marked malarial cachexia; and who, just after my entrance into her room, was seized with a violent convulsion, which soon passed off, leaving her in a semi-comatose condition. Thirty grains of chloral were administered as soon as she was able to swallow. Immediately upon sufficient dilatation of the os uteri the forceps were applied, and she was delivered of a healthy child. She had no more convulsions, and made a good recovery. The favorable result in this case is fairly attributable, I believe, to the early administration of the chloral and the prompt application of the forceps. In another case, where the convulsion occurred just after the birth of the child, thirty grains of chloral were administered; another convulsion, and one-sixth of a grain of morphia was used by hypodermic injection; no more convulsions, and the patient recovered promptly. My experience leads me to believe that puerperal convulsions are of frequent occurrence in malarial regions; probably in consequence of the blood deteriorating from the subtle poison of those regions. Now if chloral is so efficient in *curing* convulsions, might not its timely administration to women in labor prevent their occurrence?"

The doctor speaks highly of the value of chloral in producing relaxation in case of rigidity of the os uteri. He believes it contraindicated when there is organic disease of the heart or danger of pulmonary congestion. Finally, he desires to urge upon his professional brethren the importance of the employment of this agent in suitable cases.

While referring to the subject of chloral in obstetrics, we append the following inquiry from Dr. Charles B. Tydings, of Big Spring, Ky., and possibly Dr. Starley or some other of our contributors will furnish the desired information. Dr. T.'s inquiry is, "Does the hypnotic effect of chloral, when administered as an anæsthetic in parturition, extend to the child also?" He then states that in the only case under his observation it was given in fifteen-grain doses every twenty minutes for an hour and twenty minutes. The mother was unconscious at the birth of her child; and both mother and child fell into a profound slumber of eighteen hours' duration. Is it usual for such results to occur?—*American Practitioner.*

